# Long-Term Health-Related Quality of Life and Clinical Outcomes in Patients with β-Thalassemia after Splenectomy

**DOI:** 10.3390/jcm12072547

**Published:** 2023-03-28

**Authors:** Giovanni Caocci, Olga Mulas, Susanna Barella, Valeria Orecchia, Brunella Mola, Alessandro Costa, Fabio Efficace, Giorgio La Nasa

**Affiliations:** 1 Hematology Unit, Businco Hospital, Department of Medical Sciences and Public Health, University of Cagliari, 09121 Cagilari, Italy; 2 Pediatric Clinic, Thalassemia and Rare Diseases, Pediatric Hospital “Microcitemico A. Cao”, 09121 Cagliari, Italy; 3 Health Outcomes Research Unit, Italian Group for Adult Hematologic Diseases (GIMEMA) Data Center, 00161 Rome, Italy

**Keywords:** β-thalassemia, splenectomy, health-related quality of life

## Abstract

Few data are available on the efficacy and safety of splenectomy in patients with transfusion-dependent Beta-Thalassemia Major (β-TM) and on its impact on a patient’s health-related quality of life (HRQoL). We examined the long-term HRQoL of adult patients with β-TM in comparison with those treated with medical therapy by using the Medical Outcomes Study 36-Item Short-Form Health Survey (SF-36). We also evaluated the safety and efficacy of splenectomy. Overall, 114 patients with a median age of 41 years (range 18–62) were enrolled in this cross-sectional study. Twenty-nine patients underwent splenectomy (25.4%) at a median age of 12 years (range 1–32). The median follow-up after splenectomy was 42 years (range 6–55). No statistically significant differences were observed in any of the scales of the SF-36 between splenectomized and not-splenectomized patients. The majority of surgical procedures (96.6%) were approached with open splenectomy. Post-splenectomy complications were reported in eight patients (27.5%): four overwhelming infections, three with pulmonary hypertension, and one with thrombosis. A significantly higher prevalence of cardiovascular comorbidities (58.6 vs. 21.2%, *p* < 0.001) and diabetes (17.2 vs. 3.5%, *p* = 0.013) was observed in splenectomized patients. These patients, however, required fewer red blood cell units per month, with only 27.6% of them transfusing more than 1 unit per month, compared with 72.9% of the not-splenectomized group. Overall, our data suggest that physicians should carefully consider splenectomy as a possible treatment option in patients with β-TM.

## 1. Introduction

Beta-Thalassemia Major (β-TM) is a genetic disorder characterized by a reduced or absent production of the beta-globin chain in the hemoglobin (Hb) protein. Currently, thalassemias are distinguished phenotypically into transfusion-dependent (TDT) and non-transfusion-dependent (NTDT), according to the transfusion requirements [1]. In patients with β-TM, splenomegaly is a common feature following red blood cell hemolysis and extramedullary hemopoiesis [1]. Some patients may require splenectomy, with the therapeutic attempt to decrease red blood cell (RBC) transfusions and, ideally, the iron overload following the intensity of the transfusion burden.

The transfusion regimens adopted in the last decades, aimed at keeping pre-transfusion Hb concentrations above 9–10.5 g/dL, have notably reduced the incidence of splenomegaly in patients with β-TM. Recent guidelines suggest caution before adopting splenectomy, due to the possible increased risk of venous thrombosis and pulmonary hypertension, besides the overwhelming infections after splenectomy [2,3,4]. This surgical procedure should be avoided in children less than 5 years because of a greater risk of fulminant sepsis, and the main indications are limited only to an increased blood requirement that prevents adequate control with iron chelation treatment; a hypersplenism with cytopenia causing clinical problems; symptomatic splenomegaly or a massive increase in spleen dimensions with a possible risk of rupture [3]. Due to these restricted indications, the probability of patients undergoing splenectomy is substantially decreased for patients with β-TM born in the last decades, dropping from 57% to 7% for those born in the 1960s and in the 1990s, respectively [5]. Conflicting data have been reported on the efficacy of spleen removal in prolonging RBC survival and ultimately reducing the need for blood transfusions and heart and liver iron overload [6,7,8,9,10,11]. A recent review of randomized and quasi-randomized controlled studies was unable to find good-quality evidence about the efficacy of splenectomy for treating β-TM [12]. Among the parameters used to evaluate the patient’s wellness in a chronic disease such as β-TM, health-related quality of life (HRQoL) plays a key role [13,14,15]. HRQoL is generally conceptualized as a multidimensional construct referring to patients’ perceptions of the impact of disease and treatment on their physical, psychological, and social functioning and well-being [16]. To the best of our knowledge, very few studies have assessed the HRQoL of patients with β-TM after splenectomy, and these have mainly included pediatric populations or those who had a short follow-up [17,18,19]. We hypothesized that the HRQoL of splenectomized patients with β-TM was not better than that of patients who received standard medical treatment, possibly because of a greater burden of comorbidities developed over the long-term period. Therefore, we analyzed the HRQoL of adult patients with β-TM after a long follow-up post-splenectomy compared with the HRQoL of patients treated with standard medical therapy. We also evaluated the safety and efficacy of splenectomy in patients who underwent this procedure.

## 2. Materials and Methods

### 2.1. Study Design and Data Collection

In this cross-sectional study, patients with β-TM, who attended the Thalassemia and Rare Diseases Clinic of the Microcitemico Hospital in Cagliari (Italy), were included. All patients were initially contacted by mail or telephone to inform them of the study’s purposes. The following clinical and sociodemographic information was collected for the purpose of this study: employment status and type, education level, living arrangements, marital status, number of children, and comorbidities at the time of the survey. Further clinical data were derived from hospital medical records: age at splenectomy, type of splenectomy (open or laparoscopy), cholelithiasis and concurrent cholecystectomy, vaccination pre-splenectomy, and complications post-splenectomy (bleeding, thrombosis, overwhelming infections, pulmonary hypertension). A decrease of <30% in the annual RBC transfusion requirement was described as “no response”, a decrease of ≥30% was a “partial response”, and no need for transfusions was described as a “complete” response [7].

The study was performed in accordance with the Declaration of Helsinki, and it was approved by the Ethics Committee of Cagliari (authorization number: PG/2021/14301). All patients provided written informed consent.

### 2.2. Health-Related Quality of Life Assessment

All patients completed the Medical Outcomes Study 36-Item Short-Form Health Survey (SF-36) (version 1), a well-established generic HRQoL measure [20,21] that was available in Italian [22]. This measure evaluates the following eight HRQoL domains: physical functioning (PF), role limitations as a result of physical functioning (RP), bodily pain (BP), general health (GH), vitality (VT), social functioning (SF), role emotional functioning (RE), and mental health (MH). In addition, two higher-order component scores can be derived from these scales (that is, the physical component score and the mental component score); however, these were not considered for the purpose of this analysis. 

### 2.3. Statistical Analysis

The patients’ characteristics are reported for the overall population and by group (splenectomy vs. no splenectomy) as the median and range or the counts and frequencies according to the type of variable considered. The Wilcoxon rank sum test and Chi-square test were used to test for differences in the patient characteristics between the two groups accordingly. The differences in the mean scores and corresponding 95% confidence intervals (C.I.) of the eight SF-36 scales between splenectomized patients and those having received medical therapy were calculated. Comparisons between the two groups were adjusted by a multivariable regression model, including the following factors: sex, age, the highest level of education (a university degree or higher vs. lower), living arrangements (living alone vs. living with someone), ferritin level and comorbidity. An eight points difference on the eight SF-36 scales was considered a minimal important difference (MID). A score difference at least equal to a MID was considered a clinically meaningful difference [23,24]. All statistical tests were two-sided with a type I error α = 0.05.

## 3. Results

Between July 2020 and July 2021, 114 patients were enrolled in the study and completed the HRQoL survey. The median age at the time of the survey was 41 years (range 18–62). The median age of the splenectomized group was higher (48 vs. 39 years); however, this difference was not statistically significant.

The median follow-up after splenectomy was 42 years (range 6–55). The sociodemographic and clinical characteristics of patients by splenectomy status are shown in Table 1.

Twenty-nine patients underwent splenectomy (25.4%). No differences were found between the group of splenectomized and not-splenectomized patients in terms of sociodemographic variables, such as gender, level of education, employment status, living arrangements, and marital status. Splenectomized patients required fewer RBC units per month, with only 27.6% of them transfusing more than 1 unit per month compared with 72.9% of the not-splenectomized group (*p* < 0.001). Splenectomized patients showed a higher prevalence of more than three long-term comorbidities (79.3 vs. 43.5%, *p* = 0.001) (Table 1). A significantly higher prevalence of cardiovascular comorbidities (58.6 vs. 21.2%, *p* < 0.001) and diabetes (17.2 vs. 3.5%, *p* = 0.013) was observed in splenectomized patients. HCV and HBC infection rates were similar between the two groups, as well as osteoporosis and secondary tumors. The median ferritin serum level was significantly lower in the splenectomy group (705.6 vs. 984.4 mcg/L, *p* = 0.018).

### 3.1. HRQoL of Splenectomized Compared to Not-Splenectomized Patients

**Figure 1:**
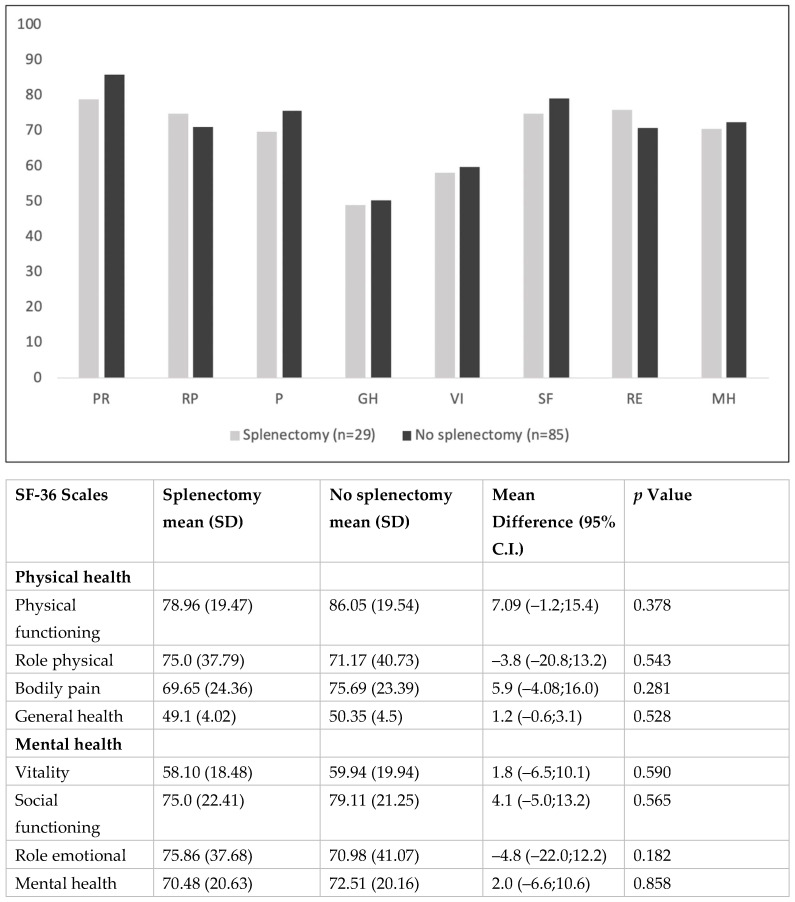
Graphical comparison of SF-36 scales mean scores between splenectomized and not-splenectomized β-TM patients. List of abbreviations: PF, physical functioning; RP, role physical; *p*, bodily pain; GH, general health; VI, vitality; SF, social functioning; RE, role emotional; MH, mental health.

The adjusted mean score differences of the eight scales of the SF-36 between splenectomized and not-splenectomized patients are depicted in Figure 1.

No statistically significant differences were observed across all the physical and mental health-related domains. The differences in mean scores and corresponding 95% CIs of the SF-36 physical health-related domains were: PF, Δ = 7.09; 95% C.I., −1.2 to 15.4, *p* = 0.378; RP, Δ = −3.8; 95% C.I., −20.8 to 13.2, *p* = 0.543; BP, Δ = 5.9; 95% C.I., −4.08 to 16, *p* = 0.281; GH, Δ = 1.2; 95% C.I., −0.6 to 3.1, *p* = 0.528). Regarding the mental health-related domains, we observed the following mean score differences: VT, Δ = 1.8; 95% C.I., −6.5 to 10.1, *p* = 0.59; SF, Δ = 4.1; 95% C.I., −5 to 13.2, *p* = 0.565; RE, Δ= −4.8; 95% C.I., −22 to 12.2, *p* = 0.182; MH, Δ = 2; 95% C.I., −6.6 to 10.6, *p* = 0.858.

### 3.2. Safety and Efficacy of Splenectomy and Clinical Outcomes

Table 2 shows data on the safety and efficacy of splenectomy. The median age at splenectomy was 12 years (range 1–32). The majority of surgical procedures (96.6%) were approached with open splenectomy, and four patients (13.8%) required a concurrent cholecystectomy. Post-splenectomy complications were reported in eight patients (27.5%). Four patients reported overwhelming infections, three patients reported pulmonary hypertension, and one patient reported thrombosis. Overall, 53.2% of splenectomized patients reported a reduction in the RBC requirement by ≥30% or a transfusion-free status.

## 4. Discussion

Our study provides novel information on the long-term HRQoL of patients with β-TM who underwent splenectomy (a median time since the surgical procedure of 42 years).

We did not observe differences in the physical and mental health-related domains in comparison with the not-splenectomized β-TM patients. The effective reduction of the transfusion burden registered after splenectomy in our cohort of patients was not associated with an improvement in the HRQoL. This outcome could be explained by recent findings indicating that, in splenectomized patients suffering from other benign hemopathies, fatigue could be exacerbated by the absence of an important immune-regulating organ, such as the spleen [25,26]. Additionally, we observed that splenectomized patients had a high comorbidity burden, with 79% of them reporting more than three comorbidities, thereby possibly impacting their HRQoL.

HRQoL in TDT is generally reported lower in comparison with the healthy population because of the transfusion dependency and related iron chelation therapy that would affect patients’ HRQoL in both physical and psychological domains, causing a high perceived burden of disease due to lifespan treatments [27]. 

There is a paucity of HRQoL data after splenectomy in β-TM patients. The HRQoL measured with the PedsQL 4.0 questionnaire [28] was reported to be lower in 7 TDT children from Sri Lanka who underwent splenectomy (compared to those who did not), in particular, regarding their psychological health dimensions, and emotional and social functioning [18]. The PedsQL 4.0 parent-reported reports were collected from parents of 49 TDT splenectomized children in Bengali, obtaining lower scores in the total summary scale in comparison with the group without surgical treatment [17].

The SF-36 questionnaire was used in a cohort of 64 splenectomized patients from Saudi Arabia, reporting significantly worse mental health scores, particularly in the vitality scale, compared to the not-splenectomized TDT patients [19]. 

The secondary objective of our study was to evaluate the safety and efficacy of splenectomy. Since the probability of undergoing splenectomy in β-TM for those born in the last decades has remarkably reduced, few data are available on the role of this surgical treatment regarding efficacy and safety [5]. Splenectomy should be avoided in children less than 5 years and limited to those with an increased blood requirement or hypersplenism with cytopenia or massive splenomegaly, with a possible risk of rupture [3,4]. In addition, peri-operative complications after splenectomy, such as a higher risk of cardiovascular complications and sepsis, have been reported [2,3,4]. This is the main reason why the benefits and risks of splenectomy should be carefully weighed before initiating this surgical treatment [2]. Nevertheless, splenectomy remains an “old weapon” [29] in the armamentarium for treating TDT patients, and clinical data on splenectomized patients have been reported from a cohort of patients treated worldwide, with contrasting results. Tugberk et al. recently reported data on 39 cases of splenectomy in Turkish patients with TDT (the mean age at splenectomy was 11 years), with a mean follow-up from the time of splenectomy of 21 years [7]. They observed a decrease of 30% or more in the annual blood requirement (defined as a partial response) in only 32% of them, with a high incidence of peri-operative complications (thrombosis in 5% of patients, infections in 28% and pulmonary hypertension in 10% of them). Data stemming from 127 TDT Italian patients confirmed that, in 64 splenectomized patients, the severity of the annual transfused blood volume was significantly lower [8]. The same research group previously demonstrated a beneficial role of splenectomy on the parameters of iron balance [30]; however, despite being regularly transfused, splenectomized patients were more frequently affected by extramedullary hematopoiesis [31]. A significant, sustained fall in annual blood transfusion requirements was also reported as a post-splenectomy outcome in a cohort of 40 Indian TDT patients [6]. Dhanya et al. published data on a broad cohort of 1064 Indian TDT, including 109 splenectomized patients, with the primary aim of detecting any difference in life expectancy between the two approaches: no statistical difference was found in the 25-year survival [10]. A study from Thailand showed that 56 splenectomized TDT patients had a significantly higher risk of pulmonary hypertension and hypogonadism [11]. A possible role of splenectomy in improving the allogeneic hematopoietic stem cell transplantation (HSCT) outcome has also been explored. Although faster neutrophil engraftment was observed, this did not produce significantly better overall survival rates after HSCT and did not provide any advantages [9,15,32].

In our cohort, 96% of splenectomies required an open surgical approach. Post-splenectomy complications were reported in 27.5% of patients: overwhelming infections (13.8%), pulmonary hypertension (10.3%) and thrombosis (3.4%).

Regarding the long-term comorbidities, we found a higher prevalence of cardiovascular comorbidities in patients who underwent splenectomy. The mechanisms underlying the association between splenectomy and cardiovascular events are not clear. Splenectomy favors a procoagulant state in patients with chronic hemolysis [33], increases the platelet count and hematocrit, and determines the persistence of procoagulant-damaged erythrocytes and platelet-derived microparticles [34,35]. 

We also found a significantly higher association between splenectomy and diabetes. Bazi et al. reported splenectomy as a risk factor for diabetes mellitus prevalence in a cohort of 148 TDT Iranian patients, with a HR of 4.3 for diabetes among splenectomized patients [36]. In trauma patients after surgery, those who received a splenectomy had a two-fold higher risk of diabetes than those without a splenectomy after a 3-year follow-up period [37]. It has been reported that human adult spleens present a reservoir of multilineage adult stem cells that may act as precursors for the β -islet secretory cells in the pancreas [38]. Moreover, the spleen plays an important role in the neurohumoral control of inflammation that is mediated by the vagus nerve, which regulates insulin production [39].

The efficacy of splenectomy on the transfusion burden was generally positive, with more than half of the patients reporting a reduction in the RBC requirement of ≥30% (Table 2), although the serum ferritin value was not significantly affected.

Our study has several limitations. Owing to the cross-sectional design of the study, we could not rely on the baseline HRQoL values of our patient population. Additionally, the patients were recruited from one center, thereby limiting the generalizability of our findings. Furthermore, given the exploratory nature of the study, our results should not be interpreted as confirmatory. Lastly, we acknowledge that the general HRQoL measure used in our study may not fully capture the key HRQoL aspects of our patients. However, we also observe that it has been widely used in previous research on patients with β-TM [40]. 

The strength of our study is that it represents one of the first reports on a long-term HRQoL in β-TM patients who underwent splenectomy (with a median of more than 40 years since the surgical procedure).

In conclusion, our results suggest that the long-term HRQoL of splenectomized patients was similar to that reported by the not-splenectomized patients. However, despite a lower burden of annual transfusion requirement, we found that splenectomy was associated with a higher prevalence of cardiovascular diseases and diabetes. Our data suggest that any potential benefit in splenectomized patients’ HRQoL, possibly generated by a lower transfusion burden, is, however, compromised by a higher prevalence of long-term comorbidities. Future research should evaluate the role of new erythropoiesis stimulators in potentially improving the HRQoL and reducing RBC transfusions. In addition, the HRQoL in thalassemia patients should be assessed with a generic questionnaire in association with specific thalassemia tools, such as the TranQoL tool [40,41]. Indeed, exploring the specific dimensions of medication compliance, treatment-related stress, and the impact of thalassemia on the family could provide further insight into the burden of this chronic disease on the patient’s HRQoL [42].

Our findings suggest that physicians should carefully consider splenectomy as a possible treatment option for patients with β-TM. 

## Figures and Tables

**Table 1:** Patient characteristics of splenectomized and not-splenectomized β-TM patients.

	Total		Splenectomy		No Splenectomy		*p*
**No. of patients. No. (%)**	114	(100)	29	(25.4)	85	(74.6)	
**Median age at survey. years (range)**	41.4	(18–62)	47.5	(21–60)	39.3	(18–62)	0.164
**Gender. No. (%)**							0.638
Males No. (%)	43	(37.7)	12	(41.4)	31	(36.5)	
Female No. (%)	71	(62.3)	17	(58.6)	54	(63.5)	
**Level of education. No. (%)**							0.932
High school degree or higher. No. (%)	69	(60.5)	18	(62.1)	51	(60)	
Compulsory School. No. (%)	45	(39.5)	11	(37.9)	34	(40)	
**Employment status. No. (%)**							0.912
Employed No. (%)	53	(46.5)	13	(44.8)	40	(47.1)	
Unemployed No. (%)	61	(53.5)	16	(55.2)	45	(52.9)	
**Living arrangements. No. (%)**							0.487
Living alone No. (%)	24	(21.1)	5	(17.2)	19	(22.4)	
Living with family/partner No. (%)	90	(78.9)	24	(82.8)	66	(77.6)	
**Marital Status. No. (%)**							0.656
Unmarried No. (%)	53	(46.5)	15	(51.7)	38	(44.7)	
Married/with partner No. (%)	61	(53.5)	14	(48.3)	47	(55.3)	
**Comorbidities. No. (%)**	107	(93.9)	23	(79.3)	79	(92.9)	0.484
**>3 comorbidities** **. No. (%)**	60	(52.6)	23	(79.3)	37	(43.5)	0.001
**CV comorbidities** **. No. (%)**	35	(30.7)	17	(58.6)	18	(21.2)	0.000
**Diabetes. No. (%)**	8	(7)	5	(17.2)	3	(3.5)	0.013
**Osteoporosis** **. No. (%)**	80	(70.2)	23	(79.3)	57	(67.1)	0.213
**HBV infection. No. (%)**	3	(2.6)	1	(3.4)	2	(2.4)	0.750
**HCV infection** **. No. (%)**	69	(60.5)	17	(58.6)	52	(61.2)	0.808
**Secondary tumors** **. No. (%)**	6	(5.2)	2	(6.9)	4	(4.7)	0.658
**Ferritin (** **mcg/L)** **. mean (range)**	913.5	(128–4620)	705.6	(250–2677)	984.4	(128–4620)	0.018
**Iron chelation. No. (%)**							0.097
No No. (%)	3	(2.6)	2	(6.9)	1	(1.2)	
Yes No. (%)	111	(97.4)	27	(93.1)	84	(98.8)	
**Oral iron chelation** **. No. (%)**	106	(93)	26	(89.7)	80	(94.1)	0.417
**Subcutaneous iron chelation** **. No. (%)**	25	(21.9)	7	(24.1)	18	(21.2)	0.739
**Transfusional need** **. No. (%)**							0.970
No No. (%)	3	(2.6)	2	(6.9)	1	(1.2)	
Yes No. (%)	111	(97.4)	27	(93.1)	84	(98.8)	
**RBC units per month** **. No. (%)**							0.000
0–1 No. (%)	44	(38.6)	21	(72.4)	23	(27.1)	
2–3 No. (%)	70	(61.4)	8	(27.6)	62	(72.9)	

List of abbreviations: CV, cardiovascular; HBV, hepatitis B virus; HCV, hepatitis C virus; RBC, red blood cells.

**Table 2:** Safety and efficacy of splenectomy in patients with β-TM.

**No. of patients, No. (%)**	29	(100)
**Median age at splenectomy, years (range)**	12.3	(1–32)
**Median follow-up after splenectomy, years (range)**	42	(6–55)
**Gender, No. (%)**		
Males No. (%)	12	(41.4)
Female No. (%)	17	(58.6)
**Splenectomy procedure, No. (%)**		
Open splenectomy, No. (%)	28	(96.6)
Laparoscopic splenectomy, No. (%)	1	(3.4)
**Concurrent cholelithiasis, No. (%)**		
Yes No. (%)	23	(79.3)
No No. (%)	6	(20.7)
**Concurrent cholecystectomy, No. (%)**		
Yes No. (%)	4	(13.8)
No No. (%)	25	(86.2)
**Pre-splenectomy vaccinations, No. (%)**		
Yes No. (%)	3	(10.3)
No No. (%) *	26	(89.7)
**Post-splenectomy complications, No. (%)**	8	(27.6)
Bleeding, No. (%)	0	(0)
Thrombosis, No. (%)	1	(3.4)
Infections, No. (%)	4	(13.8)
Pulmonary hypertension, No. (%)	3	(10.3)
**Annual transfusional need post-splenectomy** **, No. (%)**		
Reduction of RBC < 30%, No. (%)	13	(44.8)
Reduction of RCB ≥ 30%, No. (%)	14	(48.3)
Transfusion free, No. (%)	2	(6.9)

List of abbreviations: RBC, red blood cells. * All patients with no or uncertain vaccination before splenectomy were vaccinated in the following years.

## Data Availability

The medical charts and database are available from the Pediatric Clinic, Thalassemia and Rare Diseases, Pediatric Hospital “Microcitemico A. Cao”, Cagliari, Italy, upon reasonable request.
